# *Moringa oleifera* seed ethanol extract and its active component kaempferol potentiate pentobarbital-induced sleeping behaviours in mice via a GABAergic mechanism

**DOI:** 10.1080/13880209.2022.2056207

**Published:** 2022-05-19

**Authors:** Wei-Liang Liu, Bai-Fen Wu, Jian-Hua Shang, Xue-Feng Wang, Yun-Li Zhao, Ai-Xiang Huang

**Affiliations:** a Yunnan Engineering Research Center of Fruit Wine, QuJing Normal University, QuJing, People’s Republic of China; b Yunnan University of Business Management, Kunming, People’s Republic of China; c State Key Laboratory of Phytochemistry and Plant Resources in West China, Kunming Institute of Botany, Chinese Academy of Sciences, Kunming, People’s Republic of China; d College of Food Science and Technology, Yunnan Agricultural University, Kunming, Yunnan, China; e Key Laboratory of Medicinal Chemistry for Natural Resource, Ministry of Education and Yunnan Province, School of Chemical Science and Technology, Yunnan University, Kunming, People’s Republic of China

**Keywords:** Sedative-hypnotic, Cl^–^ influx, GABA_A_ receptors, GABA_A_-ergic systems

## Abstract

**Context:**

*Moringa oleifera* Lam. (Moringaceae) (MO) is an important food plant that has high nutritional and medical value. However, there is limited information on whether its seeds can improve sleep.

**Objective:**

This study investigated the effects of MO seed ethanol extracts (EEMOS) on sleep activity improvement and examined the underlying mechanisms.

**Materials and methods:**

Male ICR mice were placed into six groups (*n* = 12) and treated as follows: Control (sodium carboxymethyl cellulose, 20 mL/kg), estazolam tablets (2 mg/kg), EEMOS (1, 2 g/kg) and kaempferol (1, 2 mg/kg). These samples were successively given intragastric for 14 d. Locomotor activity assay, pentobarbital-induced sleeping and pentetrazol-induced seizures tests were utilized to examine the sedative-hypnotic effects (SHE) of EEMOS.

**Results:**

Compared with the control group, the results revealed that EEMOS (2 g/kg) and KA (2 mg/kg) possessed good SHE and could significantly elevate the levels of γ-aminobutyric acid and reduce the levels of glutamic acid in the mouse hypothalamus (*p* < 0.05). Moreover, SHE was blocked by picrotoxin, flumazenil and bicuculline (*p* < 0.05). EEMOS (2 g/kg) and KA (2 mg/kg) significantly upregulated the protein expression levels of glutamic acid decarboxylase-65 (GAD_65_) and α_1_-subunit of GABA_A_ receptors in the hypothalamus of mice (*p* < 0.05), not affecting glutamic acid decarboxylase-67 (GAD_67_) and γ_2_-subunit expression levels (*p* > 0.05). Additionally, they cause a significant increase in Cl^-^ influx in human cerebellar granule cells at a concentration of 8 µg/mL (*p*  <  0.05).

**Discussion and conclusions:**

These findings demonstrated that EEMOS could improve sleep by regulating GABA_A_-ergic systems, and encourage further clinical trials to treat insomnia.

## Introduction

In mammals, sleep is regarded as a vital process, as it occupies one-third of human-being’s life span. Sleep-related disorders influence a large number of general populations worldwide (Singh and Zhao 2017). Insufficient sleep leads to daytime dysfunction, drowsiness, decreased immunity, anxiety and depression (Heffner et al. 2012). Insomnia, one of the most commonly reported sleep disturbances, is induced by some external and internal factors such as increased work competition, urbanisation, irregular daily habits and endocrine dyscrasia (van Dalfsen and Markus 2018; Van Laake et al. 2018). According to an epidemiological study, ∼10%–30% of the population suffer from insomnia (Erden et al. 2016). Generally, pharmacological treatment is the common conventional method used to treat insomnia. It has been reported that some classical medications such as benzodiazepine (BZD) and non-benzodiazepine (NBZD) can exert sedative-hypnotic effects (SHE) to combat sleep disorders. However, long-term use of these compounds produces many side-effects including muscle relaxation, amnesia, drug dependence, tolerance and cognitive impairment.

Therefore, it is necessary to seek natural products in order to avoid the drawbacks mentioned above. It is well known that some natural functional foods such as whole grains, Lingzhi, Maca, *Panax*, Kiwi fruits, Walnut and milk can improve sleep in many people (Daliri and Lee 2015). Several studies have indicated that the intake of less functional food or diet can cause insomnia (Gangwisch et al. 2020). Although the association between natural products and sleep is clear, the functional and molecular processes underlying sleep improvement still remains elusive and requires further clarification.

*Moringa oleifera* Lam. (Moringaceae) (MO) is a fast-growing perennial and angiosperm species that is widely distributed in the tropics or subtropics; it is recognized by several other names such as drumstick tree, horseradish tree and malunggay (Alegbeleye 2018). Also, it is commonly consumed as a vegetable and used as a medicine because of its highly valuable nutritional and functional active components (Mulugeta and Fekadu 2014). Due to its strong vitality, potential medicinal values and socioeconomic advantages, it has been grown in many subtropical and tropical countries around the world. It is initially introduced to Yunnan Province, China from India for local cultivation in 1960s, and soon it has spread to Taiwan, Fujian, Guangdong, Guangxi province and other regions (Posmontier 2011).

MO is a miraculous and multipurpose tree that possesses highly digestible proteins, potassium, calcium, irons, vitamins, essential amino acids and antioxidants (Toppo et al. 2015). It is also suitable to fight malnutrition, especially among infants and nursing mothers in many developing nations where malnourishment is a major concern (Taha et al. 2015). Almost all parts (e.g., leaves, bark, flowers, fruit, seeds, tubers and roots) of MO are potentially useful and have important nutrients (Leone et al. 2016). Some studies have revealed that the seeds are probably the most valuable part of the plant, and contain a significant percent of high quality oil and a series of bioactive agents such as flavonoids (Liu et al. 2020), proteases (Mustafa et al. 2021), alkaloids, phenolics, saponins, steroids, terpenoids, carotenoids (β-carotene), vitamins, fibres and 4-[(α-l-rhamnosyloxy)benzyl]isothiocyanate (Falowo et al. 2018).

Recently, some studies have reported that using MO seeds (MOS) powder in bread, biscuit, noodles, cake and dairy products can improve nutritional values (Aluko et al. 2013; Alam et al. 2014). MO leaf has some resemblance of nutrient substance with that of seeds, and after fermentation, the levels of essential amino acids, polyunsaturated fatty acids and phytochemical concentration of MOS powder are increased, especially when applied in fermented dairy products, without affecting the colour of the foods (Ijarotimi et al. 2013). Additionally, MO seeds have been consumed in daily foods by the Malaysian population. Besides its applications in foods as mentioned above, MOS is highly effective on the treatments of flocculation, diabetes (Bao et al. 2020), cancer (Elsayed et al. 2015), hepatic injury (Saini et al. 2016), immune dysregulation (Anudeep et al. 2016), oxidative stress and convulsions (González-Trujano et al. 2018).

Many natural products have been used to regulate mental disorders in human beings (Pluskal and Weng 2018). All these products contain alkaloid, and they interact strongly with the neuroreceptors of the central nervous system (CNS; Yan et al. 2015). Nevertheless, flavonoids are rich in almost all terrestrial plants and are consumed as daily food by humans. It has become clear that they may also play a key role in neuroreceptor systems of the brain, thereby preventing neurodegenerative diseases such as Alzheimer’s disease and Parkinson’s, anxiety disorders, depression and insomnia (Jäger and Saaby 2011). it has been denoted that the blood-brain barrier (BBB) is mainly formed by brain capillary endothelial cells and functions as a physical and metabolic barrier (Pardridge et al. 1994). Furthermore, the low levels of vesicular transport and tight junctions of brain endothelial cells and high metabolic activities limit the transcellular transport processes (Teng et al. 2017), so some small and large water-soluble compounds cannot cross the BBB. Therefore, the CNS activity is considered as a prerequisite for some compounds to traverse the BBB and enter the CNS. Both *in vitro* and *in vivo* evidence shows that absorbed oral flavonoids can pass the BBB, exhibiting various effects on the CNS (Kavvadias et al. 2004).

According to many studies, MO possesses several flavonoids such as quercetin, epicatechin, catechin, gallocatechin, kaempferol (KA), rutin, chlorogenic acid, ellagic acid and ferulic acid that can confer lots of health benefits (Onsare and Arora 2015). Among these compounds, KA that is observed in MOS and predominantly involves flavonols exhibits multiple therapeutic properties such as anti-inflammation (Tang et al. 2015), neuroprotection (Filomeni et al. 2012), antioxidation (Devi et al. 2015), antianxiety (Aguirre-Hernández et al. 2010) and anticancer activities (Kim et al. 2016).

Additionally, KA has been found in many edible plants such as chives, cabbage, amaranth, tea, cucumber and turnip greens, and it is also common in some traditional medicine [i.e., *Albizia lebbeck* (L.) Benth. (Fabaceae), *Aloe vera* L. *(*Asphodeloideae)*, Cissussicyoides, Tilia americana* L. (Malvaceae)*, Lycium chinense* L. (Solanaceae)*, Houttuynia cordata* Thunb. (Saururaceae) and *MO*]. In the meantime, a large number of epidemiological studies have shown possible association between intake of KA-rich foods (i.e., broccoli, onions, kale, coffee, tea, strawberries, apple juice and orange juice) and a reduced risk of several disorders including cardiovascular diseases, cancer and mental disorders (Calderón-Montaño et al. 2011).

Nowadays, MOS has become a unique plant resource in Yunnan Province due to its specific properties like convenience of collection, low cost and high production. All these advantages contribute to a solid foundation for scientific research. Several reports have demonstrated health benefits of MOS. One of the major bioactive compounds, KA, possesses a wide range of pharmacological and physiological activities as described above. However, it is necessary to seek new functional activities and applications in functional foods and pharmaceuticals.

Although many studies have elucidated a variety of physiological functions of MOS, no studies have investigated the effect of it on sleep. Interestingly, KA is a well-known flavonoid component, which is identified in MOS and can prevent neurodegenerative diseases, anxiety disorders and even convulsions, indicating its potential effect as a depressant. Due to lack of reports on combating insomnia with MOS, the sleep improvement effects of its biologically active agent KA and the underlying mechanism were further investigated.

## Materials and methods

### Plant material and ethanol extraction

The cleaned and dried seeds of MO were purchased from Yunnan Tianyou Biotechnology Inc. (Kunming City, Yunnan Province, China) in May 2019, and a variety of PKm2 were cultivated by them. It was identified by Prof. Huang (College of Food Science and Technology, Yunnan Agricultural University, Yunnan, China). The voucher specimen (No.14CS8077) was deposited in Kunming Institute of Botany, Chinese Academy of Sciences. These seeds were then pulverized and 1000 g powder were extracted successively with 70% ethanol (10 L × 3) for 72 h by maceration at room temperature (25 °C) (Cárdenas-Rodríguez et al. 2014). The ethanol extract of MO seeds (EEMOS) was filtered with cotton wool and concentrated using rotary evaporator to a certain volume until it had no alcohol smell. After crude extraction, EEMOs were stored in sterile containers at 4–8 °C in the refrigerator for further use.

### Determination of chemical characteristics

To determine the chemical characteristics of EEMOS, samples were analysed with a Agilent 1100 high performance liquid chromatography (HPLC) system (Agilent Technologies, Inc., Santa Clara, CA, USA), which included an autosampler, a quaternary solvent delivery system and a DAD detector. Sample separation was carried out on Diamonsil C18 column (250 × 4.6 mm, 5 μm, YMC Inc., Japan). KA and other components of EEMOS were detected at 30 °C. DAD detector was set at 254 or 280 nm. Gradient elution was performed with solvent A (0.1% formic acid in water) and solvent B (acetonitrile) for 25 min at a flow rate of 1.0 mL/min (Xu et al. 2019). The injection volume was 5 µL. After completing injection, the system was equilibrated for 7 min before the next injection. The quantification assay was performed in triplicate.

### Animals

Male ICR mice weighing 18–22 g were purchased from the SJA Laboratory Animal Inc. (Hunan Province, China, Licence No. SYXK 2016-002), and housed in a controlled environment at a constant temperature (24 °C ± 1 °C) with a relative humidity of 50% ± 10% and a 12 h light/dark cycle. All mice had free access to water and food. The behavioural tests were performed during 8:00 a.m. to 3:00 p.m. in a quiet place in the above-mentioned conditions. All mice were randomly divided into different groups (*n* = 12 per group).

All animal studies (including the mice euthanasia procedure) were done in compliance with the regulations and guidelines of Yunnan Agricultural University Institutional Animal Care (the protocol approval number: SYXK 2013-0004.), and conducted according to the Association for Assessment and Accreditation of Laboratory Animal Care and the Institutional Animal Care and Use Committee guidelines. Every effort was made to minimize the number of animals used and their suffering.

### Reagents

Pentobarbital sodium (PENT), flumazenil (FMZ), muscimol (MUS), bicuculline (BIC), sodium carboxymethyl cellulose (CMC-Na), polyoxyethylene sorbitan monooleate (Tween-80, TW) and picrotoxin (PIC) were purchased from Sigma-Aldrich Chemical Inc. (St. Louis, MO, USA). Pentylenetetrazole (PTZ) was obtained from National Institute for Food and Drug Control (Beijing, China), and estazolam tablets (EST) were bought from Huazhong Pharmaceutical Inc. (Wuhan, Hubei, China). Mouse γ-amino butyric acid (GABA) and glutamic (Glu) ELISA kit were purchased from Jiancheng Biotech Inc. (Nanjing, China). KA (≥95%) was purchased from Coolaber Biotech Inc. (Beijing, China). Specific rabbit polyclonal antibodies against GABA_A_ receptors subunits (α_1_, γ_2_) or glutamate decarboxylase (GAD_65/67_) and the corresponding conjugated antirabbit immunoglobulin G-horseradish peroxidase were obtained from Abcam Inc. (Cambridge, UK). Human cerebellar granule cells (HCGC) were purchased from the BeNa Culture Collection Inc. (Beijing, China). Dulbecco’s Modified Eagle’s Medium (DMEM) and foetal bovine serum (FBS) were purchased from GIBCO Inc. (Grand Island, NY, USA). The Cl^-^-sensitive fluorescence probe (SFLUOP) and *N*-(ethoxycarbonylmethyl)-6-methoxyquinolinium bromide (MQAE) were obtained from Invitrogen Inc. (Carlsbad, CA, USA). All other chemical agents used in the study were of analytical grade.

### Locomotor activity measurement

**Figure 1.**
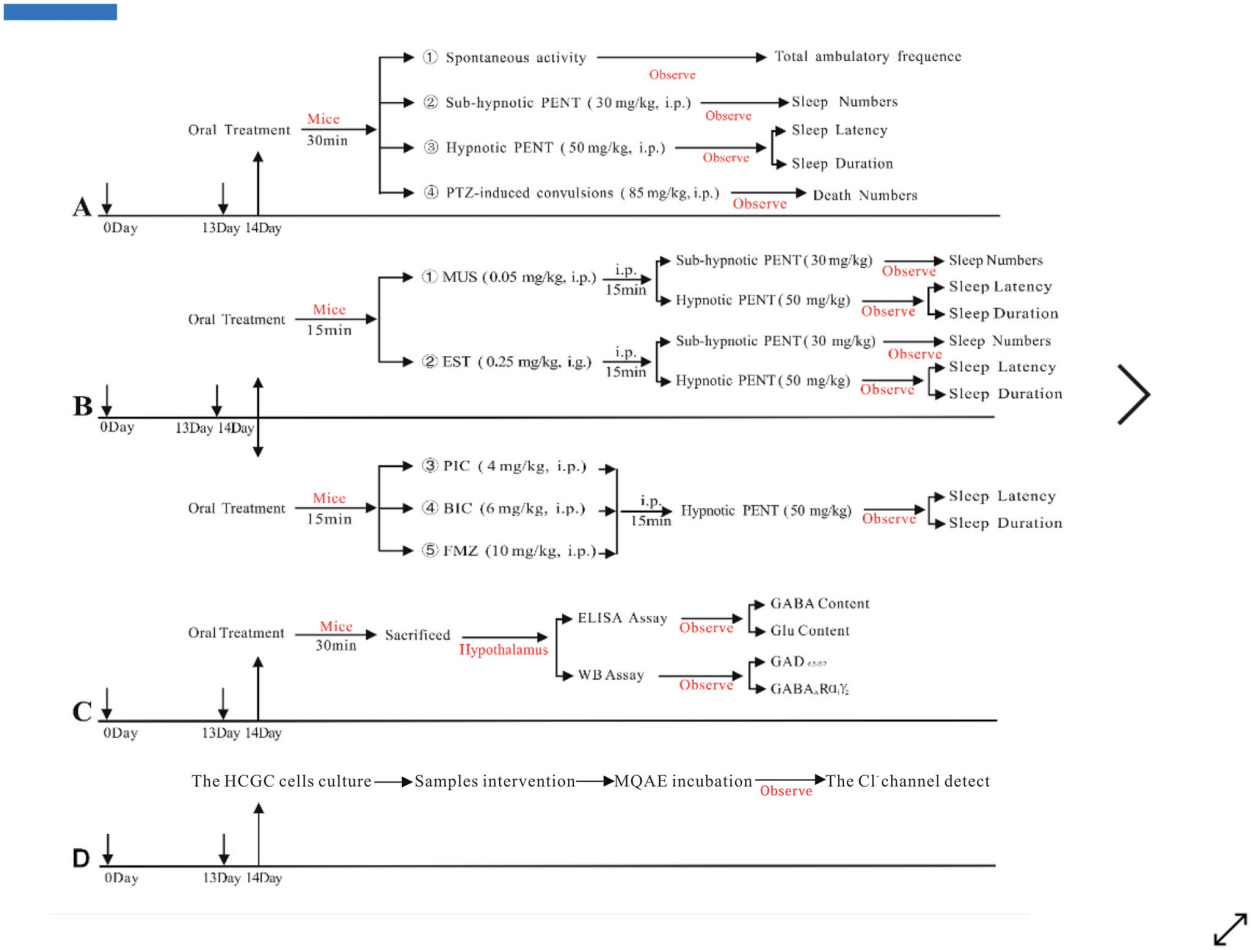
Scheme of the experimental procedure. (A) The SHEs evaluation of EEMOS. (B) The sleep improvement mechanism of EEMOS was investigated by GABA_A_ receptor synergic and antagonistic tests. (C) EEMOS promoting sleep test. (D) Intracellular Cl^–^ influx detection in HCGC cells.

The spontaneous activities of mice were detected by an autonomous activity instrument, ZZ-6 locomotor activity tester (Taimeng Software Inc., Chengdu, Sichuan, China; Zhu, Di et al. 2018; Zhu, Noviello et al. 2018). The mice were individually placed in the activity cages for 30 min after the last oral treatment of test samples, and then were acclimated to the environment for 3 min. After that, the horizontal locomotor activity was measured and recorded automatically for 5 min by microcomputer control system. The experimental protocol is shown in Figure 1(A).

### PENT-Induced sleeping test

In this experiment, 30 min after the last oral administration, the mice were intraperitoneally (IP) injected with subhypnotic (30 mg/kg, 10 mL/kg) or hypnotic (50 mg/kg, 10 mL/kg) PENT. The number of sleeping mice within 10 min was recorded, and the percentage of mice at the onset of sleeping was calculated. Additionally, the sleep latency and sleep duration were also recorded (Fajemiroye et al. 2014). The experimental protocol is shown in Figure 1(A).

### PTZ-Induced seizure test

This animal model was established by IP injection of PTZ (85 mg/kg, 10 mL/kg) 30 min after the last oral administration of test samples. Following the treatment with PTZ, mice were observed for 15 min for the occurrence of convulsions. After that, the parameters such as death number and mortality were measured based on anticonvulsant properties. The percentage of mortality protection was then calculated. The experimental protocol is shown in Figure 1(A).

### GABA_A_ receptor synergy test

EST (0.25 mg/kg, 20 mL/kg) and MUS (0.05 mg/kg, 10 mL/kg) were two specific GABA_A_ receptor agonists used for evaluating the involvement of GABA_A_ receptor in the SHE of EEMOS and KA. EST is a BZD site specific agonist and MUS is a GABA site specific agonist (Kwon, Hong et al. 2017). Mice were intragastrically (IG) or IP injected with the agonists 15 min after the administration of EEMOS and KA, respectively. The experimental protocol is shown in Figure 1(B).

### GABA_A_ receptor antagonists experiment

PIC (4 mg/kg, 10 mL/kg), which is a non-competitive channel blocker, targets the GABA_A_ receptor chloride channels. BIC (6 mg/kg, 10 mL/kg) is a light-sensitive competitive antagonist, and FMZ (10 mg/kg, 10 mL/kg) is a BZD site antagonist that forms a complex with GABA_A_ receptor. All these three specific GABA_A_ receptor antagonists were used to verify GABA_A_ receptor participation in the SHE of EEMOS and KA (Cho et al. 2012). The mice were IP injected with the antagonists 15 min after the administration of EEMOS and KA. The experimental protocol is shown in Figure 1(B).

### Brain GABA and glu contents measurement

To verify the effects of EEMOS and its bioactive agents on brain GABA and Glu levels, mice were sacrificed after the treatments with the test samples or CMC-Na solution. The brains were then rapidly maintained on an ice pad, and the hypothalamus was isolated, weighted and stored at −80 °C until extraction (Jeon et al. 2015). The levels of GABA and Glu in the brain were detected according to the manufacturer’s instructions of the detection kits. The experimental protocol is shown in Figure 1(C).

### Western blot (WB) analysis

The WB assay was performed as described previously with a slight modification (Zhang et al. 2019). Briefly, the tissue samples of the mice hypothalamus were isolated from the brain after oral administration of test samples, and the total proteins were obtained by using RIPA lysis buffer and protease inhibitors, which were immediately added before use. The immunoreactive bands were measured by enhanced chemiluminescence WB detection system and analysed with imageJ software. The experimental protocol is shown in Figure 1(C).

### Cell culture

The HCGC cell line were obtained from American Type Culture Collection and purchased from BeNa Culture Collection (Beijing, China). HCGC cells were plated (1.5 × 10^4^ per 0.2 mL) in 96-well microplates that were coated with poly-d-lysine and were attached to the bottom of the plate. They were cultured in high-glucose DMEM supplemented with 10% FBS and 1% penicillin-streptomycin solution in a humidified 5% carbon dioxide (CO_2_)/95% atmosphere at 37 °C. To promote the survival of HCGC cells, a high concentration of potassium was necessary to induce persistent cell membrane depolarisation. After culturing for 14 days, these cells expressed functional GABA_A_ receptors.

### Measurement of Cl^–^ Influx in cerebellar granule cells

Several studies have demonstrated that in the physiological state, the extracellular and intracellular chloride concentrations should be ∼130 mmol/L (mM) and 10 mM, respectively (Winsky-Sommerer 2009). When GABA or GABA_A_ receptor agonists bind with GABA_A_ receptor, Cl^–^ channels are open and the Cl^–^ influx flows into the cell along the concentration gradient. Furthermore, the cell membrane hyperpolarization exerted the sedative, antianxiety and antiepileptic effects (Jonsson Fagerlund et al. 2010). MQAE, which is a Cl^–^-SFLUOP, is used for detecting intracellular Cl^–^. When it interacts with Cl^-^, its FLUO is quenched instantaneously (Yan et al. 2015). Thus, it acts as a FLUO indicator in an ion concentration-dependent manner.

To evaluate whether EEMOS and KA could make Cl^–^ channels open in HCGC cells, MQAE was used to estimate the Cl^–^ influx according to a previously described method by West and Molloy (1996), with a slight modification. Cells were incubated in 10 mM MQAE dissolved in Cl^–^-containing buffer (pH = 7.4, HEPES: 10 mM, d-glucose: 10 mM, K_2_HPO_4_: 2.4 mM, KH_2_PO_4_: 0.6 mM, MgSO_4_: 1.2 mM and NaCl: 130 mM) and cultured for 45 min at 37 °C. Then, the cells were washed thrice in Cl^–^-free buffer before treatment with EEMOS (2, 4, 8 µg/mL), KA (2, 4, 8 µg/mL) or PENT (10 mM) dissolved in Cl^–^-containing buffer for 5 min.

The buffer was then replaced by 3.5 mM tributyltin chloride and 2.5 mM nigericin sodium prior to the addition of 10.5 M KSCN and 1.75 mM valinomycin to quench the intracellular MQAE fluorescence. Repetitive FLUO measurements (excitation wavelength, 320 nm; emission wavelength, 460 nm) were recorded instantly using a Spectra-Max-i3x Multi-Mode Microplate Reader. Data are presented as the relative FLUO *F*_0_/*F*, where *F*_0_ is the FLUO without Cl^–^ ions and F is the FLUO as a function of each sample. The *F*_0_/*F* ratio was directly proportional to the intracellular Cl^–^ concentration. The experimental protocol is shown in Figure 1(D).

### Statistical analysis

Statistical analysis was performed using SPSS software and GraphPad Prism 5. Data from experimental analyses were expressed as means ± standard error of the mean (SEM). For multiple comparisons, data were analysed by one-way analysis of variance (ANOVA) followed by Student-Newman-Keuls test. For the test with a subthreshold dose of PENT and antiseizure test, χ^2^ test was used to compare the number of mice that fell asleep or died. *p* < 0.05 was considered to be statistically significant.

## Results

### EEMOS component analysis

**Figure 2.**
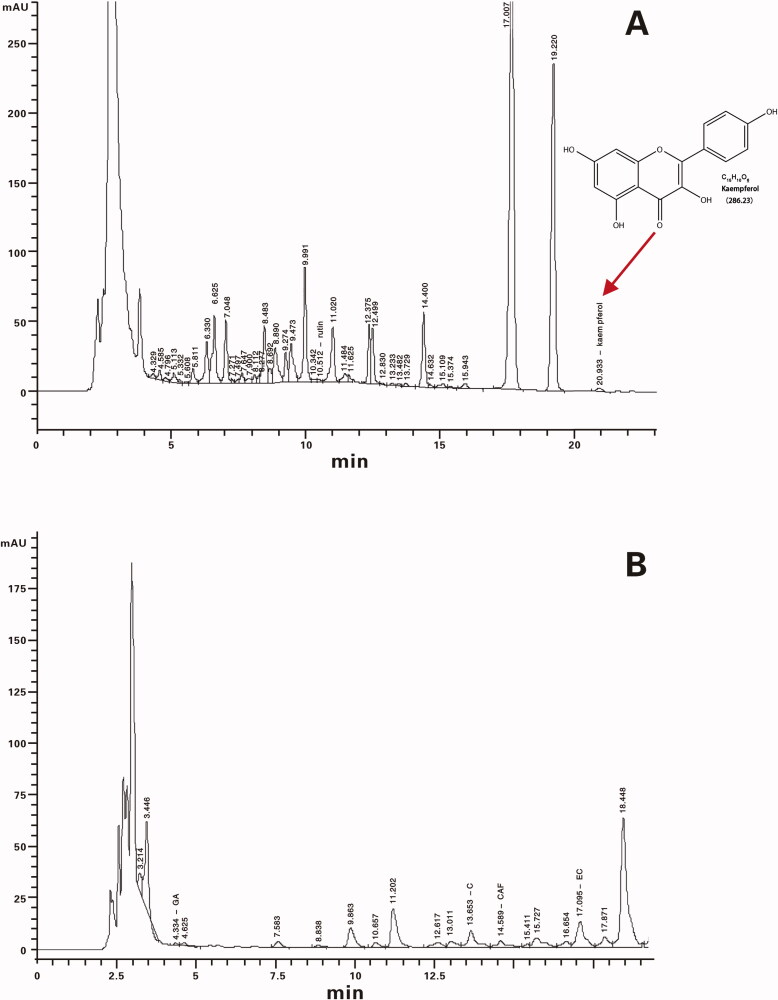
HPLC chromatograms of KA, rutin (A) and phenolic compounds (B) from EEMOS.

The contents of phenolic compounds and flavanoids (KA and rutin) in EEMOS were analysed by HPLC and the chromatograms were shown in Figure 2 and Table 1. In EEMOS, total concentrations of polyphenols, KA and rutin were 113.44, 2.72 and 2.05 mg/g, respectively. KA was detected at 20.933 min after injection and rutin peak was observed at 10.512 min.

**Table 1.** The amount of rutin, KA and phenolic compounds in EEMOS.

Retention time (min)	Compound	Concentration (mg/g)
10.512	Rutin	2.05
20.933	KA	2.72
4.334	GA	0.97
13.653	C	43.3
14.589	CAF	5.91
17.095	EC	61.43
22.864	ECG	1.83
–	EGCG	ND

Note: C, catechin; CAF, caffeine; EC, epicatechin; ECG, epigallocatechin; EEMOS, ethanol extract of MO seeds; EGCG, epigallocatechin gallate; GA, gallocatechin; KA, kaempferol; ND, not detected.

These data suggested that EEMOS contained KA, rutin and phenolic compounds, showing a positive effect on sleep induction.

### Effect of EEMOS on autonomic activities in mice

**Figure 3.**
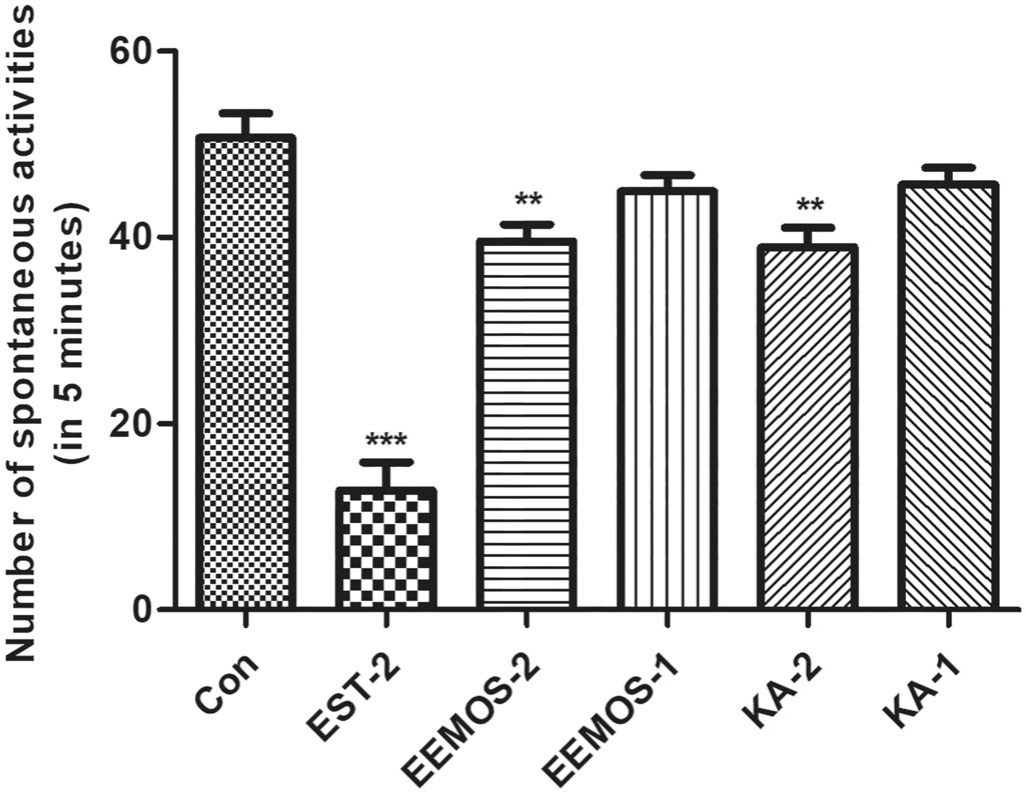
Effects of oral administration of EEMOS on locomotor activity in mice. Each column is presented as means ± SEM (*n* = 12). The mice were acclimated to the environment for 3 min in the machine, 30 min after IG administration of EST (2 mg/kg), EEMOS (2, 1 g/kg) and KA (2, 1 mg/kg). The significant effects of the compounds were assessed using ANOVA followed by Student-Newman-Keuls test. **p < 0.01, ***p < 0.001 compared to control (0.5% CMC-Na consisting 2% TW). Con: control; EEMOS: ethanol extract of MO seeds; EST: estazolam tablets; KA: kaempferol; SEM: standard error of the mean.

Compared to the control group (CG, 50.67 ± 9.24), the results demonstrated that all mice in the sample treatment groups demonstrated various degrees of reduced spontaneous activity in a dose-dependent manner, including EEMOS (2 g/kg, 39.58 ± 6.19, *p* < 0.01), EEMOS (1 g/kg, 44.92 ± 6.04, *p* > 0.05), KA (2 mg/kg, 38.92 ± 7.28, *p* < 0.01) and KA (1 mg/kg, 45.67 ± 6.31, *p* > 0.05) groups (Figure 3). Moreover, locomotory activity was significantly inhibited in the positive EST group (2 mg/kg, 12.83 ± 10.46, *p* < 0.001, Figure 3). These findings indicated that EEMOS and its bioactive components exerted inhibitory effects on CNS in a dose-response manner.

### Potentiation of PENT-induced sleeping

**Figure 4.**
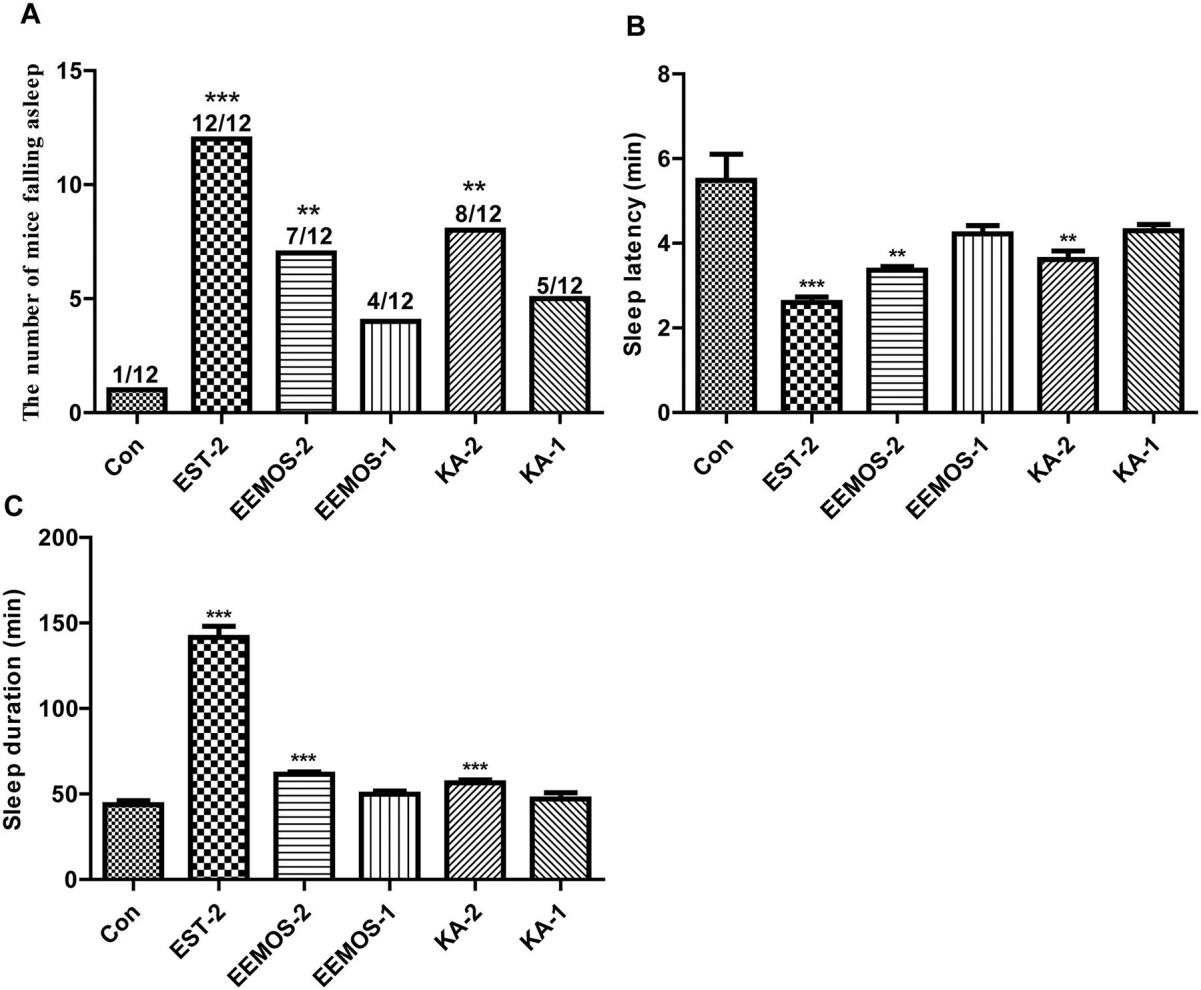
Effects of EEMOS on hypnotic response in PENT-treated mice. All values are presented as means ± SEM (*n* = 12). (A) The number of mice falling asleep. (B) Sleep latency. (C) Sleep duration. *p < 0.05, **p < 0.01, ***p < 0.001 compared to Con (0.5% CMC-Na consisting 2% TW).

To evaluate the hypnotic activity of EEMOS, a sleep test was performed on mice treated with a subthreshold dose of PENT as shown in Figure 4. Compared with CG, the results revealed significantly increased number of sleeping mice in the oral treatment group, including EEMOS (2 g/kg, *p* < 0.01) and KA (2 mg/kg, *p* < 0.01) groups, indicating that EEMOS and KA played a role in hypnosis.

The positive EST group also had significantly increased number of sleeping mice (2 mg/kg, *p* < 0.001, Figure 4(A)). Furthermore, the sleep latency (Figure 4(B)) and sleep duration (Figure 4(C)) were studied in mice receiving a threshold dose of PENT. Compared with CG (5.49 ± 2.03 min), sleep latency occurred in a dose-dependent manner after the treatments with EEMOS (2 g/kg, 3.36 ± 0.3 min, *p* < 0.01), EEMOS (1 g/kg, 4.23 ± 0.64 min, *p* > 0.05), KA (2 mg/kg, 3.62 ± 0.65 min, *p* < 0.01) and KA (1 mg/kg, 4.30 ± 0.48 min, *p* > 0.05). Moreover, significantly shorter sleep latency in normal mice was seen in the positive EST group (2 mg/kg, 2.60 ± 0.43 min, *p* < 0.001).

Concurrently, compared to CG (43.69 ± 8.22 min), sleep duration occurred in a dose-dependent manner after the treatment s with EEMOS (2 g/kg, 61.56 ± 4.52 min, *p* < 0.001), EEMOS (1 g/kg, 49.88 ± 6.41 min, *p* > 0.05), KA (2 mg/kg, 56.52 ± 5.84 min, *p* < 0.001) and KA (1 mg/kg, 47.08 ± 12.20, *p* > 0.05). Significantly prolonged sleep duration in normal mice was seen in the EST group (2 mg/kg, 141.63 ± 21.30 min, *p* < 0.001). These results demonstrated that EEMOS and KA exerted synergistic effects with PENT.

### Effect of EEMOS on PTZ-induced seizure

PTZ, which is a non-competitive antagonist of GABA_A_ receptor, is usually used to induce convulsions in the animal model of epilepsy. Mice treated with PTZ (85 mg/kg, ip) experienced myoclonic jerk seizures. Thus, the antiseizure effects of EEMOS and KA were examined on a series of characteristic behavioural symptoms such as tremors of vibrissae and muscles, which were observed after PTZ injection to record death number, mortality and the percentage of mortality protection of each group.

As shown in Table 2, compared with CG, all mice administered with EST (2 mg/kg) were protected (100%) and these mice had no convulsions after PTZ injection (*p* < 0.001). The mice in the other oral treatment groups showed the effects in varied degrees against the PTZ-induced convulsions and a dose-dependent protection. Particularly, a significant reduction in the mortality rate was seen in the EEMOS (2 g/kg, 66.7%, *p* < 0.01) and KA (2 mg/kg, 25%, *p* < 0.001) groups. These results indicated that EEMOS and KA had antiseizure effects in PTZ-induced mice model.

**Table 2.** Anticonvulsant effect of EEMOS and KA on PTZ-induced convulsions in ICR mice.

Treatment	Number	Dose/kg	Death number	Mortality (%)	Mortality protection (%)
Con	12	20 mL	11	91.7	–
EST	12	2 mg	0	0	100***
the Low dose of EEMOS	12	1 g	8	66.7	27.3
the High dose of EEMOS	12	2 g	4	33.3	63.6**
the Low dose of KA	12	1 mg	7	58.3	36.4
the High dose of KA	12	2 mg	3	25	72.7***

**p < 0.01, ***p < 0.001 significant compared to Control.

Abbreviations: Con: control; EEMOS: ethanol extract of *Moringa oleifera* seeds; EST: estazolam tablets; KA: kaempferol.

Mortality (100%) = (number of mice died after convulsion/total number of mice used) × 100%; Mortality protection (%) = (the death number of blank group – the death number of experimental groups)/the death number of blank groups × 100%.

### Synergistic effect of EEMOS and MUS in PENT-treated mice

To investigate the direct relationship between the hypnotic activity of EEMOS and the GABAergic system, EEMOS with MUS was co-administered on PENT-induced sleeping mice. The pre-treatment low-dose EEMOS (1 g/kg), KA (1 mg/kg) and MUS (0.05 mg/kg) showed no effects on sleep latency and sleep duration induced by PENT (50 mg/kg). The rate of sleep onset induced by subhypnotic dosages of PENT was also unaffected.

**Figure 5.**
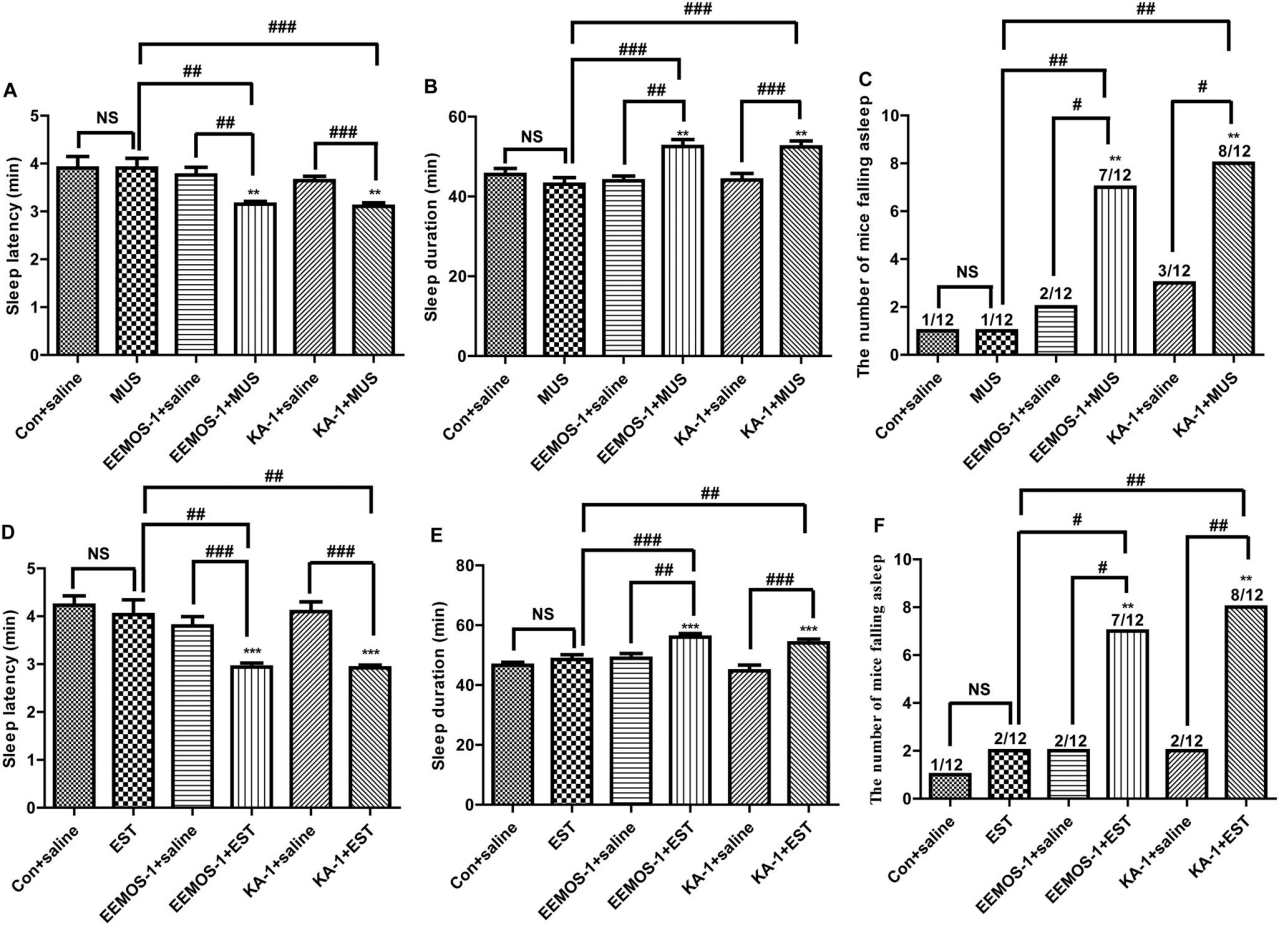
Synergic effects of EEMOS with GABA_A_ receptor agonists on hypnotic response in PENT-treated mice. Sleep latency (A), sleep duration (B) and the number of mice falling asleep (C) combined with MUS. Sleep latency (D), sleep duration (E) and the number of mice falling asleep (F) combined with EST. All data are presented as means ± SEM (*n* = 12). **p < 0.01, ***p < 0.001 compared to Con; ^#^*p* < 0.05, ^##^*p* < 0.01, ^###^*p* < 0.001 compared to agonist plus each treatment group. NS: not significant.

Surprisingly, co-administration with MUS (0.05 mg/kg) exhibited synergistic effects on decreasing sleep latency (*p* < 0.01, *p* < 0.001, Figure 5(A)) and significantly prolonged sleep duration (*p* < 0.01, *p* < 0.001, Figure 5(B)) when compared with self-control group (SCG). The co-administration also significantly increased the number of sleeping mice (*p* < 0.05, *p* < 0.01, Figure 5(C)) in mice treated with subhypnotic dosages of PENT.

### Synergistic effect of EEMOS and Est in PENT-treated mice

To verify the underlying mechanisms of the depressant activity of EEMOS, the effect of concomitant treatment of EEMOS and KA with an ineffective dose of EST (0.25 mg/kg), a well-known agonist of the GABA_A_-BZD receptor, were tested. The pre-treatment low-dose of EEMOS (1 g/kg), KA (1 mg/kg) and EST (0.25 mg/kg) showed no effects on sleep latency and sleep duration induced by PENT when administered alone. Similarly, the rate of sleep onset induced by subthreshold dosages of PENT was unaffected.

Interestingly, co-administration with EST (0.25 mg/kg) exhibited synergistic effect on decreasing sleep latency (*p* < 0.01, *p* < 0.001, Figure 5(D)) and prolonging sleep duration (*p* < 0.01, *p* < 0.001, Figure 5(E)) when compared with SCG. Co-administration also dramatically increased the number of mice sleeping (*p* < 0.05, *p* < 0.01, Figure 5(F)) treated with subhypnotic dosages of PENT.

### Effects of co-administration of EEMOS with PIC on PENT-treated mice

**Figure 6.**
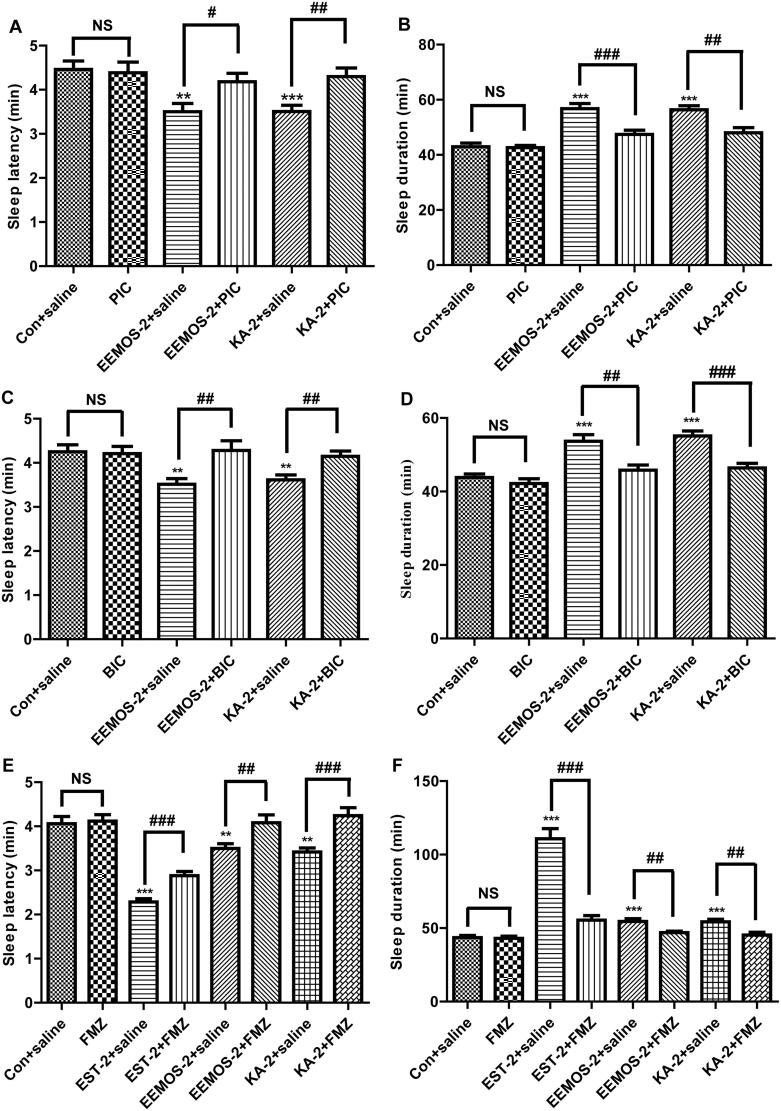
Effects of GABA_A_ receptor antagonists on hypnotic activity of EEMOS. All data are presented as means ± SEM (*n* = 12). Sleep latency and sleep duration with PIC (A and B), BIC (C and D) and FMZ (E and F). **p < 0.01, ***p < 0.001 significant compared to Con; ^#^*p* < 0.05, ^##^*p* < 0.01, ^###^*p* < 0.001 significant between antagonist treatment and non-antagonist treatment. GABA_A_: gamma-amino butyric acid type A receptor.

To demonstrate the mechanisms of hypnotic effects of EEMOS and KA, GABA_A_ receptor non-competitive inhibitor PIC was used. As depicted in Figure 6, EEMOS (2 g/kg) and KA (2 mg/kg) alone significantly shortened the sleep latency (3.50 ± 0.65 min and 3.51 ± 0.50 min, respectively), and increased the sleep duration (56.75 ± 6.56 min and 56.36 ± 5.01 min, respectively), when compared with CG (4.46 ± 0.66 min and 42.98 ± 4.66 min, respectively; *p* < 0.01, *p* < 0.001). Pre-treatment with PIC (4 mg/kg) alone showed no influence on sleep latency (Figure 6(A)) and sleep duration (Figure 6(B)) induced by PENT (50 mg/kg).

Concomitant treatment of mice with EEMOS, KA and PIC revealed that the hypnotic effect was completely antagonized by PIC (*p* < 0.05, *p* < 0.01 and *p* < 0.001, respectively) when compared with SCG. These data indicated that EEMOS might exert sedative effects via GABAergic neurotransmission.

### Effects of co-administration of EEMOS with BIC on PENT-treated mice

As many functional components for treating sleeping loss target GABA_A_ receptor, which constitutes BZD, GABA, barbiturates and nerve sterol binding sites, to treat insomnia, but the generated sedative effects of these agents could be competitively inhibited by BIC (Shi et al. 2014). Therefore, BIC (a GABA_A_ receptor antagonist) was utilized to explore the mechanism of the actions of EEMOS.

As shown in Figure 6, EEMOS (2 g/kg) and KA (2 mg/kg) alone significantly shortened the sleep latency (3.52 ± 0.44 min, 3.61 ± 0.40 min, respectively), and increased the sleep duration (53.68 ± 6.29 min, 55.12 ± 4.63 min, respectively) when compared to CG (4.25 ± 0.55 min and 43.82 ± 3.33 min, respectively; *p* < 0.01, *p* < 0.001). Based on our previous work, treatment with BIC (6 mg/kg) alone did not influence sleep latency (Figure 6(C)) and sleep duration (Figure 6(D)) induced by PENT (50 mg/kg). When mice were given EEMOS and KA with BIC, the hypnotic effect was fully blocked (*p* < 0.01, *p* < 0.001) when compared with SCG. According to these data, the tranquilising effects of EEMOS and KA might be due to their GABAergic actions.

### Effects of co-administration of EEMOS with FMZ on PENT-treated mice

GABAergic inhibition has been recognized to play a central role in the regulation of sleep and wakefulness (Savage et al. 2018), and the BZD binding site on GABA_A_ receptor is targeted by most hypnotic agents. Consequently, FMZ (the GABA_A_-BZD antagonist) was used to understand the mechanism of sedative effects of EEMOS and KA.

As shown in Figure 6, compared to CG (4.06 ± 0.56 min and 43.59 ± 4.93 min), EEMOS (2 g/kg), KA (2 mg/kg) and EST (2 mg/kg) alone remarkably decreased the sleep latency (3.50 ± 0.38 min, 3.42 ± 0.33 min, 2.29 ± 0.25 min, respectively) and increased the sleep duration (54.57 ± 6.51 min, 54.34 ± 6.02 min, 110.79 ± 23.92 min, respectively) (*p* < 0.01, *p* < 0.001). Pre-treatment with FMZ (10 mg/kg) alone did not affect sleep latency (Figure 6(E)) and sleep duration induced by PENT (Figure 6(F)). As expected, the hypnotic effect of EST was significantly inhibited by FMZ (*p* < 0.001). When mice were treated EEMOS and KA with FMZ, the hypnotic activity was completely reversed by FMZ when compared to SCG (*p* < 0.01, *p* < 0.001). These findings confirmed the possible involvement of EEMOS and KA in GABAergic system that could be interrupted by FMZ.

### The levels of neurotransmitters in the hypothalamus

GABA has been well established as the main inhibitory neurotransmitter in the brain of mammals and it can activate GABA_A_ receptors that favours sleep. Glu is an excitatory neuro-transmitter that is widely distributed in the CNS and participates in the regulation of sleep. Some articles have reported that the brain functions depending on an exquisite balance between inhibitory and excitatory neurotransmission (Möhler 2006). When this balance is interrupted and shifted pharmacologically in favour of GABAergic transmission, then sedation can be induced. In contrast, it can lead to exaggerated reactivity, arousal, anxiety, insomnia and even convulsions.

Furthermore, these two neurotransmitters play a central role in intercellular neural signal transmission, and many bioactive substances exert their hypnotic activity by regulating the levels of GABA and Glu. Several studies have demonstrated that the brain regions such as cerebral cortex, diencephalon, pons and hypothalamus demonstrate close relationship with sleep, particularly the hypothalamus, where the existence of these two neurotransmitters is rich (Li et al. 2018).

**Figure 7.**
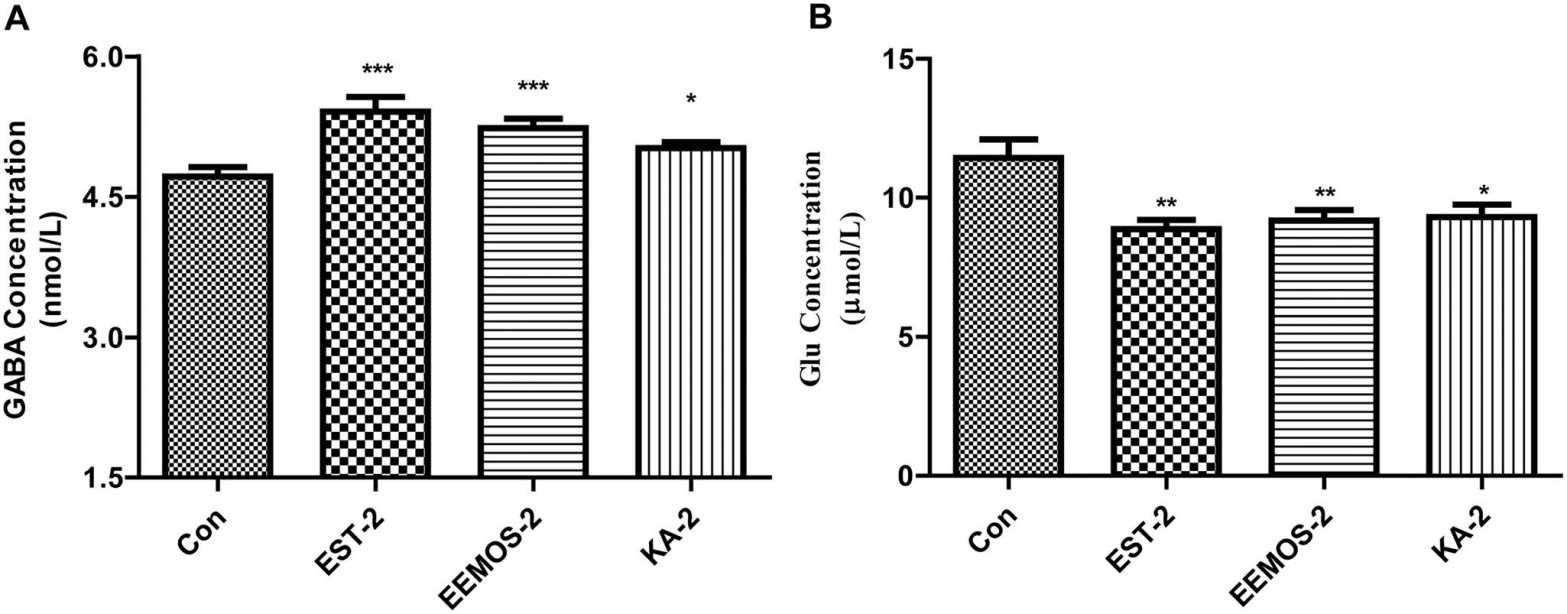
Effects of EEMOS on the levels of GABA and Glu in the hypothalamus of mice. Each column is represented as means ± SEM (*n* = 12). *p < 0.05, **p < 0.01, ***p < 0.001 compared to Con.

Consequently, the changes of GABA and Glu concentrations were determined in the hypothalamus of the mice using ELISA kit. As shown in Figure 7, compared with CG, EEMOS (2 g/kg), KA (2 mg/kg) and EST (2 mg/kg) significantly increased GABA levels (*p* < 0.05, *p* < 0.001, Figure 7(A)) and decreased Glu in the hypothalamus, respectively (*p* < 0.05, *p* < 0.01, Figure 7(B)). These data suggested that EEMOS and KA might regulate intracellular neurotransmitter concentration to develop the sedative effects.

### Effect of EEMOS on the expression of GAD_65/67_ and GABA_A_ receptor subunits

**Figure 8.**
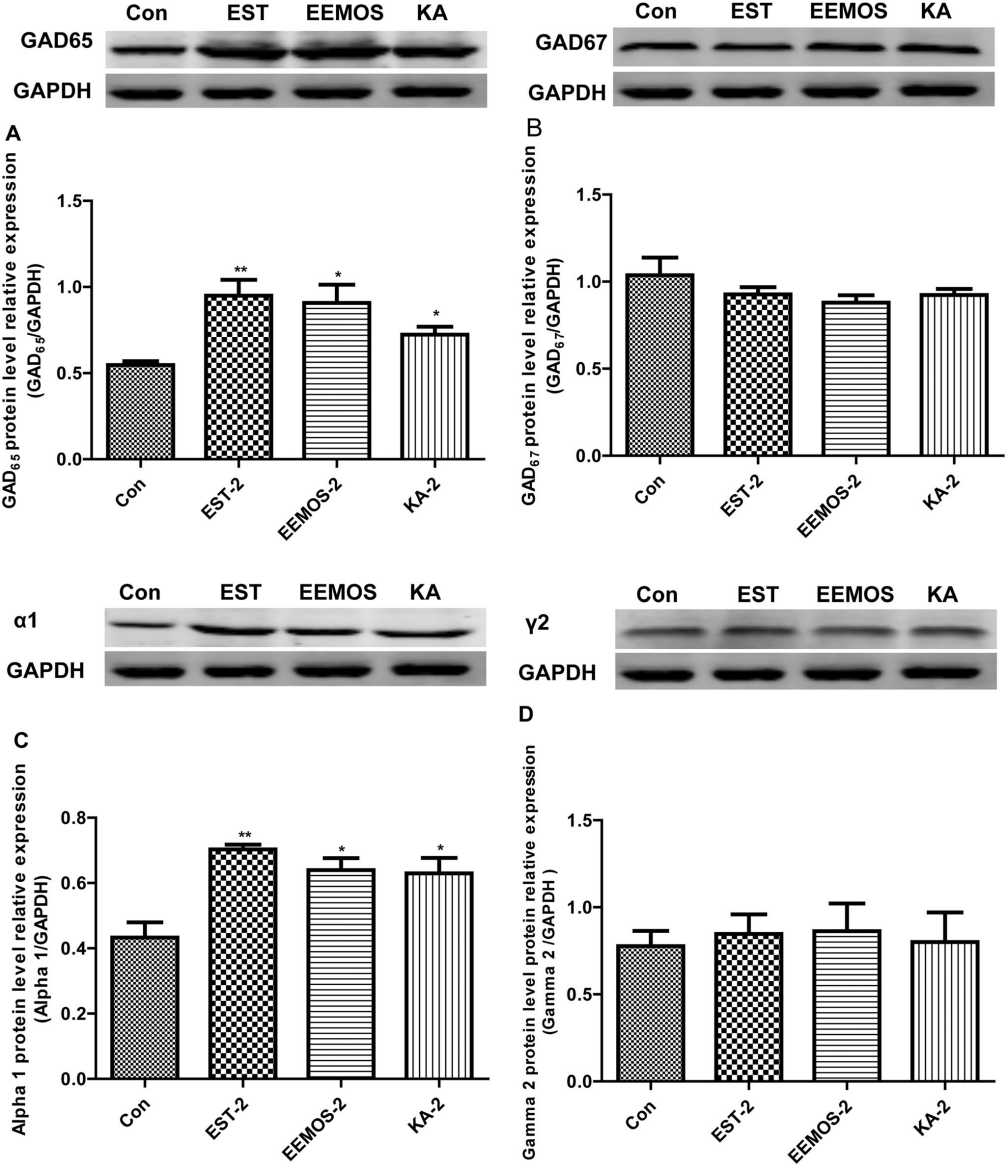
Effect of EEMOS on the expression of GABA_A_ receptor subunits and GAD_65/67_. GAPDH levels were used for normalisation of protein expression. Each column is represented as mean with SEM (*n* = 3). *p < 0.05, **p < 0.01 compared to Con. GABA_A_: gamma-Amino butyric acid type A receptor; GAD_65/67_: glutamate decarboxylase; GAPDH: glyceraldehyde-3-phosphate dehydrogenase.

To evaluate whether or not EEMOS and KA improved sleeping behaviours through the biosynthesis of GABA, the mice were given corresponding samples for 14 d to examine the activation of GAD_65/67_. Moreover, GABA_A_ receptor subunits were also assessed. As depicted in Figure 8, compared with CG, these findings indicated that EEMOS (2 g/kg) and KA (2 mg/kg) treatment increased the expression of GAD_65_ and α_1_-subunit (*p* < 0.05) (Figure 8(A,C)), but did not affect the amounts of GAD_67_, γ_2_-subunits in the hypothalamus (*p* > 0.05; Figure 8(B,D)). Additionally, EST (2 mg/kg) significantly enhanced the amounts of GAD_65_ and α_1_-subunit (*p* < 0.01), but did not influence the abundance of GAD_67_ and γ_2_-subunits.

### Effect of EEMOS on Cl^–^ influx in HCGC cells

**Figure 9.**
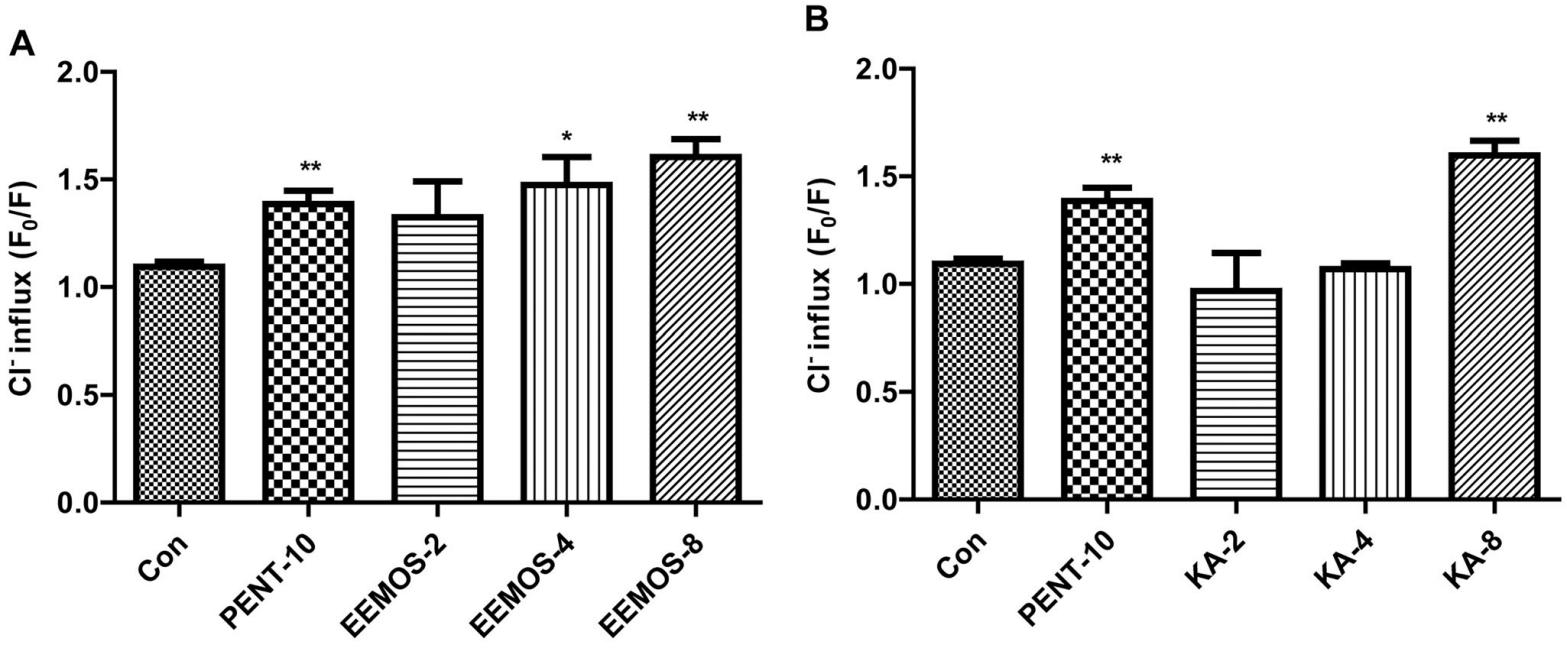
Effect of EEMOS on Cl^–^ influx in HCGC cells. Columns are represented as means ± SEM (*n* = 3); *p < 0.05, **p < 0.01 compared to Con.

In the GABAergic system, the extracellular Cl^–^ that moves into the HCGC cells was measured by MQAE. The results were denoted by relative fluorescence *F*_0_/*F* ratio, where *F* was the fluorescence as a function of each sample and *F*_0_ was the fluorescence without Cl^–^ ions. The *F*_0_/*F* ratio was directly proportional to intracellular Cl^–^ concentration. As shown in Figure 9, compared to CG, a significant increase in Cl^–^ influx was shown in the cells treated with EEMOS (4 µg/mL, *p* < 0.05; 8 µg/mL, *p* < 0.01; Figure 9(A)) and KA (8 µg/mL, *p* < 0.01, Figure 9(B)). PENT (10 mM) that acts as a positive control also increased the influx of Cl^–^ in HCGC cells (*p* < 0.01).

## Discussion

Sleep is considered as an integral mechanism to maintain the normal life in individuals. Currently, it is an essential issue for the diagnosis of certain psychiatric diseases, as they are usually observed in many patients with neurological and sleep disorders (He et al. 2020). Nowadays, insufficient sleep or poor sleep has gradually become a critical public health issue, as it is difficult for most people to achieve the recommended sleep standard (7–8 h/day; Hafner et al. 2017). Generally speaking, sleep disturbances are associated with age, but young people are facing more serious problems of insomnia currently (Cao et al. 2017). Some reports have demonstrated that sleep loss increases the risk of cancer, diabetes, obesity, depression, Alzheimer's disease and cardiovascular diseases (Benedict et al. 2020; McAlpine et al. 2019). It is well-known that sleep is a complex physiological process and is regulated by multiple neurotransmitter systems including γ-aminobutyric acid-ergic (GABAergic), 5-hydroxytryptamine-ergic (5-HTergic) and Histamin-ergic (HIS-ergic; Hong et al. 2016; Um et al. 2017). Among these neurotransmission systems, especially, GABAergic system is well-recognized due to its involvement in the activation of sleep-awake cycles.

At present, chemical drug treatments are still considered as the first choice clinically, though they often cause some undesirable adverse effects such as drug dependence, tolerance and addiction. Because of these side effects, many studies have focussed on developing more safe and unique medicines for tranquilising. Even now, when there are three generations of hypnotics developed based on the inhibitory actions of GABA_A_ receptors like barbiturates and BZDs, these drugs have exhibited good effects on the treatment of insomnia, and other new hypnotic drug research and development are still ongoing. A large number of reports have indicated that natural products not only modulate human sensations and mood, but also can relieve anxiety and improve sleep disorders (Pluskal and Weng 2018). Accordingly, much attention has been given to seeking active agents from natural products to combat insomnia.

MO is an important medicine food which possesses high therapeutic, nutritional, agricultural and socioeconomic values (Mishra et al. 2011), and all these treasure factors are attributed to its applications in modern functional food that can provide health benefits. Researchers have verified that MO is associated with several biological functions such as anti-inflammation, antioxidation, antidiabetic, anticancer, antianxiety and even anticonvulsion activities. Additionally, the folk medicine MO has been used for antianxiety and antiepilepsy attributes in Pakistan, Bangladesh and Yunnan (China) (Bakre et al. 2013). However, there are fewer studies that evaluate the sedative-hypnotic activity of its seeds. Therefore, according to our preliminary data and existing literature, we inferred that EEMOS and its active agents could affect sleep, which was further investigated in the present work. To the best of our knowledge, this was the first critical study to focus on interpreting the effects of EEMOS on sleep and the underlying mechanisms through which its hypnotic effect was generated.

Numerous studies have been conducted on flavonoids and phenolics that are major components in MOS, in particularly flavonoids. These two chemical agents belong to a group of plant secondary metabolites, which have a diphenylpropane structure and are well-known positive modulators of GABA_A_ receptors (Hanrahan et al. 2011). Flavonoid KA is a yellow compound with a low molecular weight (MW: 286.2 g/mol), which is commonly found in plant-derived foods. It could pass the BBB to regulate the CNS. It has been reported that KA isolated from *Apocynum venetum* possesses anxiolytic-like activities (Grundmann et al. 2009). However, whether KA plays a depressant-like role and is responsible for the hypnotic effects of EEMOS has not yet been identified. Therefore, the constituents of EEMOS including total polyphenol, KA and rutin were initially analysed using quantitative HPLC, which showed a value of 113.44, 2.72 and 2.05 mg/g, respectively. Additionally, most interests were focussed on the sleep time after oral administration of EEMOS and KA in PENT-induced mice. Furthermore, the possible underlying mechanisms of sleep induction via GABAergic transmission were also elucidated.

As we know, sleep is recognized as a sophisticated physiological process, which is regulated by the nervous system network and can hardly be reproduced outside the body (Fuller et al. 2006). Thus, in our current study, the SHEs of EEMOS and KA were evaluated by the spontaneous activities and PENT-induced sleep in mice (Silvestre et al. 1999), which were considered as the two classic behavioural pharmacological methods for assessing sedation-hypnotic samples. The results showed that EEMOS and KA significantly decreased the locomotor activity and augmented the hypnotic effect of PENT-induced sleeping in a dose-dependent manner. In addition, they could also remarkably increase the number of mice falling asleep after receiving a subhypnotic dose of PENT. This indicated that the excitability of the CNS was reduced, which might be attributed to the GABA_A_ receptor, similar to action of the positive EST drug. A previous study has demonstrated that the change of PENT-induced sleep time can be used as a useful tool to examine the stimulatory or inhibitory effects on CNS, especially the influences on GABAergic systems.

The anticonvulsant actions are commonly used to evaluate the effectiveness of sedative-hypnotic samples. PTZ has been shown to interact with GABA neurotransmitter to induce seizures, so some samples might exhibit good anticonvulsant effects through GABAergic pathway (Bahr et al. 2019). The present findings implied that EEMOS and KA significantly reduced the rate of PTZ-induced convulsion death in a dose-dependent manner, indicating that they could modulate the GABAergic system. Furthermore, it is necessary to verify the specific mechanisms underlying convulsion at anticonvulsant dose.

Many sleep medicines exert SHEs by targeting the GABA_A_-BZD receptors (Reite 2011). Therefore, it is necessary to evaluate whether the SHE can be blocked by the GABA_A_ receptor or the BZD receptor antagonist such as BIC, FMZ and PIC. Likewise, the synergistic effects of EEMOS and KA co-administration with GABA_A_ receptor or the BZD receptor agonists including MUS and EST were also investigated. We found that the SHE could be fully antagonized by PIC, BIC and FMZ. Additionally, the results also showed the synergistic effects of MUS and EST in PENT-treated mice. Consequently, all the above results denoted the regulatory role of EEMOS in the GABA_A_-BZD receptor complex.

In CNS, changes in the levels of neurotransmitters are marked by certain neuropharmacological actions such as sedation, hypnosis and antianxiety (Yu et al. 2019). GABA is regarded as the principal inhibitory neurotransmitter in the brain, and up to 40% of the synapses in the nervous system operate through GABA to regulate neurophysiological processes including cognition, pain, motor function, sleep and anxiety (Lancel and Steiger 1999). Glu is mainly distributed in the hypothalamus and cerebral cortex. It acts as one of the major excitatory neurotransmitters and participates in modulating neurological functions such as autonomic nervous activities, memory and sleep. The balance between these two neurotransmitter levels is usually used as an objective index to assess the excitation and inhibition of CNS function (Stephenson-Jones et al. 2020).

Thus, there is no doubt that neurotransmitters play an important role insomnia. Because hypothalamus was one of the major regulatory regions of the sleep–wakefulness cycle, the concentrations of GABA and Glu in hypothalamus were determined in the present study to explain the mechanism underlying the positive effects observed. Our findings suggested that MO and KA obviously enhanced the levels of GABA and decreased the levels of Glu in the hypothalamus, implicating that it exerted the SHEs by up-regulating GABA. Furthermore, accumulated GABA might affect various physiological functions including spontaneous locomotor behaviour and anticonvulsant actions in mice.

Glutamic acid decarboxylase (GAD_65/67_) is a rate-limiting enzyme responsible for the conversion of Glu to GABA, and plays a key role in maintaining the GABA level in the CNS (Shah et al. 2015). Thus, changes in the expression levels of this enzyme might influence GABA transmission in the brain. Reports have demonstrated that GABA_A_ receptors consisting of two α_1_, two β_2_ and one γ_2_ subunits are found in all regions of human brain (Kwon, Hong et al. 2017). Among these subunits, α_1_γ_2_ are the major subunits of GABA_A_ receptor in the hypothalamus, which is closely related to its SHEs. Besides these attributes, due to rapid inhibition of neurotransmission and the involvement in tonic inhibition, GABA_A_ receptors also act as important therapeutic targets to combat insomnia.

GAD_65/67,_α_1_ and γ_2_ subunits in hypothalamus tissues of mice are responsible for GABA production and allosteric regulation of GABA_A_ receptor. To verify the sleep-potentiating activities of EEMOS and KA via the synthesis of GABA and mediation of GABA_A_ receptors, the expression levels of these subunits were examined. Our results revealed that EEMOS and KA significantly increased the levels of GAD_65_ and α_1_ subunits in the hypothalamus of the brain, thereby activating GAD_65_ and improving GABA transmission.

It is noteworthy that GABA_A_ receptors participate in the formation of the GABA_A_-BZD receptor-Cl^–^ channel complex (Zhu, Di et al. 2018; Zhu, Noviello et al. 2018). When its agonists bind to the binding site, Cl^–^ channel opens and Cl^–^ flows into the cells, hyperpolarizing the cell membrane and reducing the neuronal excitability (Kwon, Ha et al. 2017). It is well known that GABA, pentobarbital and diazepam can directly activate Cl^–^ channel (Lambert et al. 2001). The effects of EEMOS and KA on sleep induction are associated with GABA_A_-BZD receptor-Cl^–^ channel complex, and they may act to initiate the intracellular Cl^–^ channel opening of GABA_A_ receptors. In our study, EEMOS and KA significantly increased the intracellular Cl^–^ influx in HCGC cells in a dose-dependent manner, suggesting that they acted as an allosteric activator of GABA_A_ receptors, exerting significant SHEs.

## Conclusions

MOS has high nutritional value and multiple medicinal properties. Based on its unique characteristics, it can be used as a potential resource for current functional food. Recently, MOS has been applied in various fields such as food additives, cosmetics, biofuels, lubricants and health products. Our study revealed that EEMOS could exert sedative, hypnotic and anticonvulsive effects, which might be associated with Cl^–^ channel activation in HCGC cells.

Similar to previous findings, HPLC analysis results in our study showed that MOS was rich in bioactive phytochemicals, which could confer health benefits such as improving insomnia and other mental disorders. Collectively, these results indicated that MOS possessed multifunctional active components, which might exhibit a depressant effect by activating the GABAergic system.

Furthermore, MOS could be a good candidate for the development of new functional food to help treat insomnia. Our findings might contribute to the understanding of the biological effects of EEMOS and its active agents. Further studies are necessary to confirm the involvement of 5-hydroxytryptamine-ergic (5-HTergic) system in mediating the SHE, and no efforts should be spared to seek more active agents with tranquilising features in EEMOS.
